# Therapeutic vaccination against autologous cancer stem cells with mRNA-transfected dendritic cells in patients with glioblastoma

**DOI:** 10.1007/s00262-013-1453-3

**Published:** 2013-07-02

**Authors:** Einar Osland Vik-Mo, Marta Nyakas, Birthe Viftrup Mikkelsen, Morten Carstens Moe, Paulina Due-Tønnesen, Else Marit Inderberg Suso, Stein Sæbøe-Larssen, Cecilie Sandberg, Jan E. Brinchmann, Eirik Helseth, Anne-Marie Rasmussen, Knut Lote, Steinar Aamdal, Gustav Gaudernack, Gunnar Kvalheim, Iver A. Langmoen

**Affiliations:** 1 Vilhelm Magnus Laboratory for Neurosurgical Research, Institute for Surgical Research, University of Oslo, Oslo, Norway; 2 Department of Neurosurgery, Oslo University Hospital, Postbox 4956, Nydalen, 0424 Oslo, Norway; 3 Department of Clinical Cancer Research, Oslo University Hospital, Oslo, Norway; 4 Department of Ophthalmology, Center for Eye Research, Oslo University Hospital, Oslo, Norway; 5 Department of Radiology, Oslo University Hospital, Oslo, Norway; 6 Department of Immunology, Institute for Cancer Research, Oslo University Hospital, Oslo, Norway; 7 Ex Vivo Cell Laboratory, Institute of Immunology, Oslo University Hospital, Oslo, Norway; 8 Department of Oncology, Cancer Clinic, Oslo University Hospital, Oslo, Norway; 9 Department of Cellular Therapy, Cancer Clinic, Oslo University Hospital, Oslo, Norway; 10 Cancer Stem Cell Innovation Center, Oslo University Hospital, Oslo, Norway; 11 Norwegian Stem Cell Center, Oslo University Hospital, Oslo, Norway

**Keywords:** Brain cancer stem cell, Tumorsphere, Glioblastoma, Dendritic cell, Immunotherapy, Autologous cell culture

## Abstract

**Background:**

The growth and recurrence of several cancers appear to be driven by a population of cancer stem cells (CSCs). Glioblastoma, the most common primary brain tumor, is invariably fatal, with a median survival of approximately 1 year. Although experimental data have suggested the importance of CSCs, few data exist regarding the potential relevance and importance of these cells in a clinical setting.

**Methods:**

We here present the first seven patients treated with a dendritic cell (DC)-based vaccine targeting CSCs in a solid tumor. Brain tumor biopsies were dissociated into single-cell suspensions, and autologous CSCs were expanded in vitro as tumorspheres. From these, CSC-mRNA was amplified and transfected into monocyte-derived autologous DCs. The DCs were aliquoted to 9–18 vaccines containing 10^7^ cells each. These vaccines were injected intradermally at specified intervals after the patients had received a standard 6-week course of post-operative radio-chemotherapy. The study was registered with the ClinicalTrials.gov identifier NCT00846456.

**Results:**

Autologous CSC cultures were established from ten out of eleven tumors. High-quality RNA was isolated, and mRNA was amplified in all cases. Seven patients were able to be weaned from corticosteroids to receive DC immunotherapy. An immune response induced by vaccination was identified in all seven patients. No patients developed adverse autoimmune events or other side effects. Compared to matched controls, progression-free survival was 2.9 times longer in vaccinated patients (median 694 vs. 236 days, *p* = 0.0018, log-rank test).

**Conclusion:**

These findings suggest that vaccination against glioblastoma stem cells is safe, well-tolerated, and may prolong progression-free survival.

**Electronic supplementary material:**

The online version of this article (doi:10.1007/s00262-013-1453-3) contains supplementary material, which is available to authorized users.

## Introduction

Glioblastoma is the most common primary brain tumor and unfortunately has one of the poorest prognoses of all cancers. It causes progressive cognitive and physical disability, invariably leading to death. Although contrast-enhanced MRI usually indicates a distinct tumor border, islands of tumor cells can extend far into the surrounding brain tissue, thereby precluding complete surgical resection. Standard therapy has typically consisted of surgical resection followed by radiotherapy, which generally results in a median survival of less than 1 year. Although temozolomide has recently been shown to increase progression-free survival (PFS) in a selected group by 1.9 months and median overall survival (OS) by 2.5 months compared to radiotherapy alone [1], the prognosis for glioblastoma patients has improved very little since post-operative radiotherapy became the standard of care four decades ago.

Cells possessing stem cell characteristics have been identified in a wide range of tumors [2, 3]. In normal brain tissue and in glioblastoma, stem cells were first identified by their ability to form spheres of cells in vitro [4, 5]. The sphere-forming assay has subsequently been shown to be a robust method for the isolation and expansion of glioblastoma stem cells (GSCs) [6, 7]. These cells share a number of properties with stem cells from the normal adult human brain [8], which have the ability to differentiate into multi-lineage progeny, and have the capacity to propagate the tumor upon serial xenografting [6, 9–11], thus fulfilling the criteria for classification as CSCs.

Preclinical data indicate that CSCs drive tumor growth and are resistant to current therapy [7, 12, 13]; the CSC hypothesis proposes that these cells must be eradicated to cure the cancer [2, 3]. Although widely studied in preclinical models, the clinical significance of CSCs in human tumor progression remains unclear. The presence of CSCs in melanoma has been suggested to be a result of the immune status of the xenogenic recipient [14]. However, two recent reports highlight the effect of a CSC gene signature on predicting outcomes in human leukemia [15, 16]. No such data exist for solid tumors, and the clinical utility of targeting CSCs has not yet been explored. Several of the previously identified CSC antigens (such as nestin and CD133 [17, 18] and reviewed in [19]) are shared by a range of somatic stem and progenitor cell populations in different organs. The possible adverse effects of therapeutic targeting of antigens shared by these cells and CSCs are unknown and could potentially include deleterious loss of somatic stem cell populations in rapidly repopulated tissues, such as bone marrow, epidermis, or gastrointestinal epithelium.

Dendritic cells (DCs) are the most effective antigen-presenting cells in the human immune system. We have previously treated melanoma and prostate cancer patients using DCs transfected with mRNA from allogeneic cell lines or autologous tumor bulk [20, 21]. Initially, the central nervous system was considered to be immunologically privileged due to the blood–brain barrier. More recent data, however, support a high level of cellular and molecular interaction between brain tumors and the immune system. The use of DCs to target GSCs has been explored in animal models, with superior tumor control when compared with approaches utilizing tumor bulk cells [22, 23].

In the present study, we utilized autologous DCs transfected with autologous GSC-mRNA to induce an immune response against the patient’s own GSCs. We previously demonstrated the use of mRNA-transfected DCs for the targeting of human telomerase (hTERT) and survivin for cancer immunotherapy (clinicaltrials.gov ID NCT00961844 and [24, 25]). We found increased telomerase activity in GSCs compared with somatic neural progenitor cells [11], and survivin was highly expressed in GSCs [4]. To facilitate the monitoring of induced immunity and potentially act as therapeutic targets, we combined this approach with the use of hTERT- and survivin-mRNA-transfected DCs. Our results suggest that the establishment of autologous GSC cultures under good manufacturing procedures (GMP) is feasible. We that vaccination against GSCs is safe, well-tolerated, and may prolong recurrence-free survival.

## Methods

### Patients

The study protocol was evaluated and approved by the appropriate authorities: the Norwegian Data Inspectorate, the Data Protection Official, the Regional Ethical Board, the Norwegian Medicines Agency, and the Directorate of Health. The study was listed in public clinical trial databases [http://www.clinicaltrials.gov/ (ID: NCT00846456); EudraCT number 2007-006171-37] and was performed in accordance with the Norwegian and European Union regulations and the Declaration of Helsinki. Patients were recruited at Oslo University Hospital from February 2009 until February 2010. Tissue harvesting was performed after written informed consent was obtained. Inclusion criteria were primary surgery for histologically confirmed glioblastoma, age 18–70 years, Eastern Cooperative Oncology Group (ECOG) performance status 0–1, and post-operative residual gadolinium contrast-enhancing mass size of 0–5 cm^3^. Exclusion criteria were prior neoplastic, autoimmune, or immunodeficiency diseases and the need for corticosteroids during the course of vaccination. We report on the first 11 of the 20 patients planned to be included in the protocol. Of these patients, we were unable to produce tumorspheres for one patient. Three other patients could not be weaned off corticosteroids after radio-chemotherapy and were therefore excluded from further analysis. The remaining seven patients underwent the planned regimen of vaccines. Patient characteristics are detailed in the Table 1. The primary end point of this study was the development of adverse events, while secondary end points were PFS, OS, and the presence of an induced immune response. Progression was defined either as an increase in contrast-enhancing tissue on T1-MRI without subsequent regression or the need for corticosteroids due to increasing headache or neurological deficits.

**Table 1:** Adverse events and follow-up data in seven patients treated with GSC-mRNA-transfected DC immunotherapy

Patient	Age, sex	RPA	Adverse events	Progression-free survival (months)	Overall survival (months)
#1	49, F	3	Fatigue (grade 1), anorexia (grade 1)	22	24
#5	57, M	4	Fatigue (grade 1)	29	35
#6	63, M	4	Fatigue (grade 3), pain (grade 2), anorexia (grade 1), nausea (grade 1)	10	11
#8	57, F	4	Focal epileptic seizures (grade 1), Fatigue (grade 1–2), anorexia (grade 1), constipation (grade 1)	17	25
#9	46, M	3	Fatigue (grade 1), pain (grade 1), anorexia (grade 1)	NR	NR at 30
#10	61, F	4	Fatigue (grade 1), pain (grade 1–2), anorexia (grade 1), nausea (grade 1), constipation (grade 1)	15	20
#11	52, M	4	Fatigue (grade 1), pain (grade 1)	30	34

*NR* not reached

To establish a control population, we identified 77 patients from our prospectively collected tumor database [26] who were treated from 2005 to 2008 and who matched the inclusion criteria for age, functional status, and chemo-radiotherapy treatment. Post-operative MRI volumes were available for 21 of these patients. Seven of these patients had residual tumor volume >5 cm^3^ after surgery, two had massive early progression, and two were lost to follow-up. Thus, ten highly matched patients treated prior to the initiation of the current study were compared with the seven patients treated by CSC-targeted therapy (Suppl. Table 1). The historical control patients were followed according to institutional standard protocols. MRI imaging was routinely performed 6 months after surgery or at the debut of new symptoms.

### Generation of GSC cultures

Tumor biopsies (0.3–4 ml) were mechanically and enzymatically dissociated under controlled conditions in a GMP facility and cultivated in basic fibroblast growth factor 10 ng/ml, epidermal growth factor 20 ng/ml, (both R&D Systems, Minneapolis, MN, USA), leukemia inhibitory factor 10 ng/ml (Millipore, Billerica, MA, USA), B27-supplement 1:50 (Invitrogen), penicillin/streptomycin 100 U/ml each (Lonza, Basel, Switzerland), heparin 1 ng/ml (Leo Pharma, Ballerup, Denmark), and HEPES 8 mM (Lonza) in DMEM/F12 (Invitrogen) as previously described [4, 11, 27, 28]. In culture, the cells formed spheres that were dissociated into single cells using Trypsin–EDTA and re-plated at 5 × 10^4^ cells/ml. When the spheres reached a size at which their cores turned dark (70–100 μm), the cultures were trypsinized to single cells (Suppl. Fig. 1). To confirm tumorigenicity, single-cell suspensions from tertiary tumorsphere cultures were orthotopically transplanted into severe combined immunodeficiency (SCID) mice as previously described [11, 27].

### RNA isolation and amplification

Cells were collected and dissolved in a TRIzole solution (Qiagen, Nydalen, Norway) and isolated on an RNeasy Mini column. Isolated RNA was amplified and prepared for in vitro transcription based on the procedure described by Bockowski et al. [29]. First-strand synthesis was performed by incubation with 2.5 μM first-strand primer (5′-AAGCAGTGGTATCAACGCAGAGTACT(30)VN-3′, where V is G, A, or C, and N is any nucleotide, Eurogenetec, Seraing, Belgium). To this, we added DTT, reaction buffer, dNTP mixture (Clontech, Mountain View, CA, USA), SUPERase•ln RNase inhibitor (Ambion, Austin, Tx), Superscript II Reverse Transcriptase (Invitrogen), and 2 μM T7 switch primer (5′-ACTCTAATACGACTCACTATAGGGAGAGGGCGGG-3′) (Eurogentec) for reverse transcription. Second-strand synthesis was performed using an advantage 2 PCR enzyme system (Clonetech Laboratories) with RNAse H (Ambion). PCR amplification was performed using 5′-primer (5′-GCTCTAATACGACTCACTATAGG-3′) and 3′-primer (5′-AAGCAGTGGTATCAACGCAGAGT-3′) (Eurogenetec). Amplified cDNA was purified on a MinElute column (Qiagen). In vitro transcription was performed using the T7 mMESSAGE mMACHINE large-scale transcription kit (Ambion). DNA was removed by TURBO DNase (Ambion). Amplified mRNA was purified on a MEGAclear column (Ambion). Samples were then stored at −70 °C. Aliquots of purified RNA, amplified ds-cDNA, and amplified mRNA were quantified and analyzed by gel electrophoresis on a Nanodrop 2000 (Thermo Scientific, Wilmington, DE, USA), Bioanalyzer 2100 (Agilent Technologies, Santa Clara, CA, USA), and Experion 700 systems (Bio-Rad, Hercules, CA, USA).

### DC generation

DCs were generated in a closed system using a procedure similar to that described previously [20, 21, 30]. Briefly, peripheral blood mononuclear cells (PBMCs) were harvested by leukapheresis, and monocytes were enriched by immunomagnetic depletion of T cells and B cells before being cultured for 5 days in CellGro DC medium in Teflon bags (CellGenix, Freiburg, Germany) with granulocyte–macrophage-colony-stimulating factor (GM-CSF 2,500 U/ml) (Leucomax; Schering-Plough, Kenilworth, NJ, USA) and interleukin-4 (IL-4 1,000 U/ml) (CellGenix). The immature DCs were transfected with autologous GSC-amplified mRNA (tDC) using a BTX ECM 830 square-wave electroporator (Genetronics Inc., San Diego, CA, USA). To obtain adequate control DCs for the T cell assays, a fraction of immature DCs from each patient was mock-transfected (mDC), that is, electroporated without mRNA. DCs were then cultured for two more days with IL-1b (10 ng/ml), IL-6 (1,000 U/ml), tumor necrosis factor-α (TNFα; 10 ng/ml) (CellGenix), and prostaglandin E2 (1 mg/ml) (Sigma-Aldrich). The methods and results of quality controls were similar to what was described previously [29], using a FACSscan flow cytometer (Becton–Dickinson) analysis of antihuman CD1a, CD14, CD19, HLAII (Dako Cytomation, Glostrup, Denmark), CD3, CD16/CD56, CD80, CD86 (Becton–Dickinson, San Jose, CA), CD83, CCR7, and CD209 (Immunotech, Marseilles, France) (Suppl. Fig. 2). DCs were thawed, washed, and suspended in saline, and were then brought to the patient and immediately injected intradermally. For Patient #6 and subsequent patients, RNA plasmids encoding the genes hTERT and survivin were also electroporated into separate batches of DCs to facilitate monitoring of the induced immune response by providing two defined antigens in the vaccine.

### Immune monitoring

The immune response was monitored using delayed-type hypersensitivity (DTH) monitoring and a T cell proliferation assay. PBMCs were collected and frozen at baseline and at two time points during the vaccination process as described previously [25, 30]. Thawed PBMCs collected from single patients at different time points were processed in parallel and stimulated once in vitro with peptide pools (ProImmune Ltd, Oxford, UK) or lysate at 2 × 10^6^ cells/ml in serum-free CellGro DC medium (CellGenix). On day 3, 20 U/ml IL-2 (Chiron, Trondheim, Norway) was added and cultured for a total of 10 days. T cells were seeded at 5 × 10^4^ 1:1 with irradiated (30 Gy) autologous PBMCs as antigen-presenting cells. Proliferation assays were performed in triplicate and measured at day 3 after labeling with 3.7 × 10^4^ Bq ^3^H-Thymidine (Laborel, Oslo, Norway) overnight before harvesting. The stimulatory index (SI) was defined as proliferation with peptide/lysate divided by proliferation without peptide/lysate. SI ≥2 was considered a positive response.

### Treatment and clinical follow-up

**Fig. 1:**
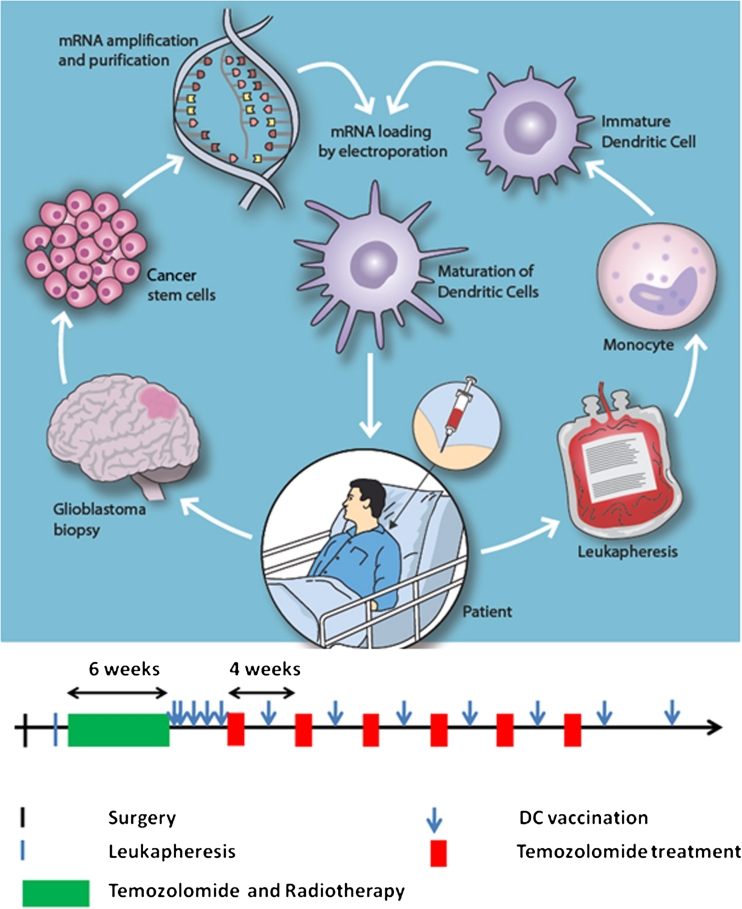
Schematic overview of the production of DCs targeting glioblastoma stem cells. *Left circle*: Tumor biopsies were collected during standard surgery. Autologous tumorsphere cultures containing brain cancer stem cells were established under GMP conditions. Upon tertiary sphere formation, RNA was purified from the cancer stem cell cultures, and mRNA was amplified using the strand switch method. *Right circle*: before the initiation of radio-chemotherapy, the patient underwent leukapheresis for harvesting of monocytes. Ex vivo-cultured autologous monocytes were then differentiated into immature dendritic cells. *Center*: The dendritic cells were transfected with autologous glioblastoma stem cell mRNA by electroporation. *Below*: Following termination of chemo-radiotherapy according to the EORTC regimen, matured dendritic cells expressing glioblastoma stem cell antigens were administered to the patient by intra-dermal injections five times for induction over the first 3 weeks and thereafter monthly for up to 18 vaccinations

All patients received post-operative chemo-radiotherapy according to the standard European Organization for Research and Treatment of Cancer (EORTC) protocols for glioblastoma treatment [1]. The patients received two vaccines during the first week after completion of combined chemo-radiotherapy, and they then received one weekly vaccine for three more weeks (Fig. 1). Following the initial 4 weeks of vaccination, patients received adjuvant temozolomide or vaccines every other week. Patients were monitored for adverse events every other week; these were scored according to the standardized common terminology criteria for adverse events v3.0 (CTCAE) according to good clinical practice (GCP) recommendations. The entire study was monitored by a GCP-qualified external monitor. A standardized ophthalmological evaluation, including optical coherence tomography, and ultrasound, was performed before vaccination and at 3-month intervals during vaccination. Brain MRIs with T1 ± gadolinium contrast, T2-, perfusion-, and diffusion-series were performed after surgery, at the start of vaccination, and every 3 months thereafter. Tumor volume assessments were made according to the RECIST version 1.1 criteria [31].

## Results

### Preclinical validation of autologous GSC cultures

We have previously worked extensively on characterizing the sphere-forming cells derived from brain tumor biopsies [11, 27, 28]. Primary tumorsphere cell cultures retain the genotype of the tumor of origin [27, 32] and maintain the ability to initiate a tumor with patient-specific characteristics upon orthotopic grafting [27, 32, 33]. To evaluate the feasibility of establishing autologous GSCs mRNA for vaccination, we performed a series of primary GSC cultures under conditions transferable to GMP conditions. We dissociated and cultivated 32 glioblastoma biopsies under serum-free conditions. Of these, 23 gave rise to tertiary tumorspheres and nine did not. The median survival of patients from whom GSC cultures could be established was 271 days, while this was not reached in patients where GSC cultures did not form (*p* = 0.027, log-rank test) (Suppl. Fig. 3). There were no significant differences between the groups regarding age, ECOG, histological diagnosis, or number of resections after the biopsy was obtained (Suppl. Table 2). Orthotopic xenografting of 2 × 10^5^ cells from ten different cell cultures, after the formation of tertiary tumorspheres, gave rise to intracerebral glioblastoma in 49 of 52 SCID mice.

### Vaccine production

An overview of the vaccine production is presented in Fig. 1. Biopsies were collected at surgery and dissociated under GMP conditions for the establishment of GSC cultures. While some cells adhered to the bottom of the dishes and developed a more differentiated phenotype, a subpopulation of cells formed secondary tumorspheres (Suppl. Fig. 1). Further culturing gave rise to tertiary spheres that were collected for the isolation of RNA. From this RNA, we produced and purified cDNA before transcription in vitro. The average amount of mRNA generated for DC transfection was 5.3 ± 2.3 × 10^2^ μg.

To facilitate monitoring for an induced immune response, DCs transfected with specific mRNA constructs encoding for hTERT and survivin were produced and administered to patients in parallel (for Patient #6 and subsequent patients).

### Immune response evaluation

The immune response was evaluated by testing the induced in vitro lymphocyte proliferation and DTH. These responses were measured based upon stimulation of pre- and post-vaccine peripheral blood T cells by GSC-lysates and hTERT- and survivin peptide mixes. In Patients #6, #8, and #11, there was not enough tumorsphere cellular material to allow for in vitro testing of the induced T-lymphocyte proliferation against GSC-lysates. In all seven patients, we found specific-induced lymphocyte proliferation upon stimulation with tumorsphere lysate, hTERT, or survivin peptides in vitro as tested during vaccination (6–9 months) and at the end of the vaccination period (9–11 months) (Table 2 and Suppl. Fig. 4). Only Patient #5 developed a positive DTH response against GSC-lysate. Throughout the vaccination period, lymphocyte levels remained low due to temozolomide treatment (Suppl. Fig. 5).

**Table 2:** Immune response evaluated by lymphocyte proliferation upon stimulation by tumorsphere lysate or a mixture of peptides from hTERT or survivin

Pat #	Baseline			6–8 months			9–11 months		
	hTERT	Survivin	TSL	hTERT	Survivin	TSL	hTERT	Survivin	TSL
1	NA	NA	1.1	NA	NA	3.8	NA	NA	2.8
5	NA	NA	0.8	NA	NA	2.76	NA	NA	0.8
6	1.3	10.6	NA	13.4	2.6	NA	4.7	4.7	NA
8	2.2	2.2	NA	12.3	18.6	NA	5.1	5.1	NA
9	1.3	1.1	0.8	1	1.4	2.7	2.3	1.7	2.7
10	2.2	1	NA	60.6	41.5	NA	3.2	1.5	NA
11	0.6	1	NA	1	1.1	NA	2.1	6.4	NA

All patients developed a significant T-lymphocytes proliferation response induced by tumorsphere lysate (TSL), hTERT, or survivin peptide in vitro. In patient #6, #8, #10, and #11, there were not enough tumorsphere cellular material to allow for testing of induced T-lymphocyte proliferation. hTERT- and survivin-mRNA-transfected DCs was added to the treatment from patient #6. NA for tests not performed

### Safety monitoring

Patients reported fatigue, anorexia, and headache graded 0–3, as detailed in the Table 1. This is comparable to the normal range of adverse events related to conventional radio-chemotherapy. Patients maintained ECOG performance status of 0–1 throughout the vaccination period. No patients developed signs of cerebral edema or autoimmune encephalomyelitis. To monitor for a possible cross-reaction against somatic neural stem cells, the patients underwent serial ophthalmological evaluations, and none of the patients developed retinal or uveal inflammation. We detected no induced autoimmune reactions against stem cells in the hematopoietic, dermal, or gastrointestinal systems.

### Evaluation of tumor progression

**Fig. 2:**
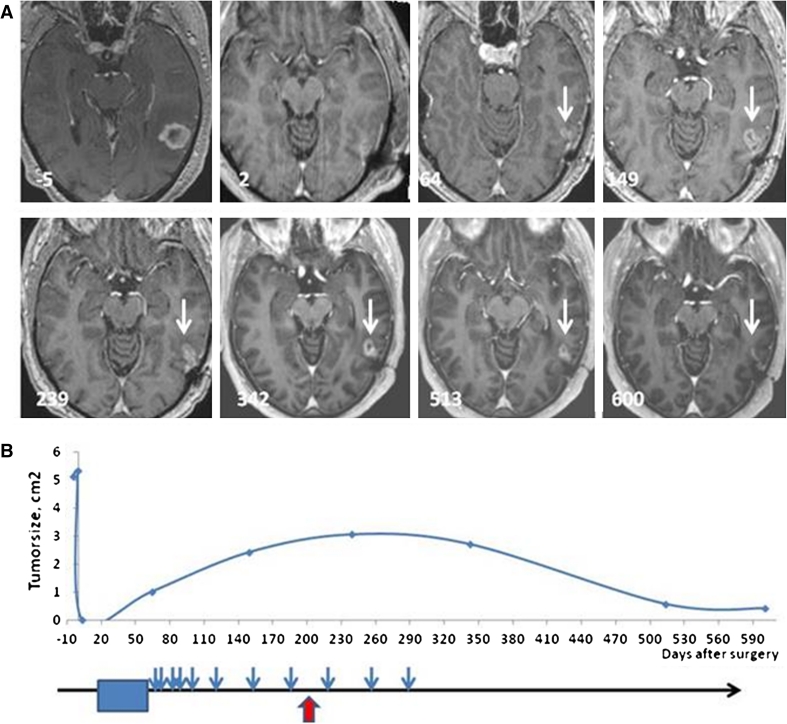
Changes in size of contrast-enhancing tumor over time. **a** Brain MRI axial T1 images after intravenous gadolinium contrast in patient #5. Days before (negative) and after surgery are noted on the MRI scans. No residual tumor was observed post-operatively (day 2), but at the end of the 6 weeks course of combined chemo/radiotherapy, a small contrast-enhancing lesion could be detected at the anterior margin of the resection cavity, as indicated with the *white arrow* (day 64). **b** Maximal area of contrast enhancement plotted against days since surgery (abscissa). *Lower part* of the figure indicates the timing of concomitant chemo-radiotherapy (*blue box*), DC vaccinations (*blue arrows*), and immune response (*red arrow*)

Tumor volume was monitored by serial brain MRIs (Fig. 2). A contrast-enhancing lesion had recurred or grown in five of the seven patients at the conclusion of radiotherapy, prior to the initiation of immunotherapy. These lesions all increased in size during the first phase of vaccination and reached a maximum mean volume of 805 mm^3^ (363–1,526 mm^3^) during ongoing vaccination. Subsequently, the contrast-enhancing lesions decreased to a minimum of 209 mm^3^ (9–452 mm^3^) after 448 days (342–568 days) (Fig. 2).

**Fig. 3:**
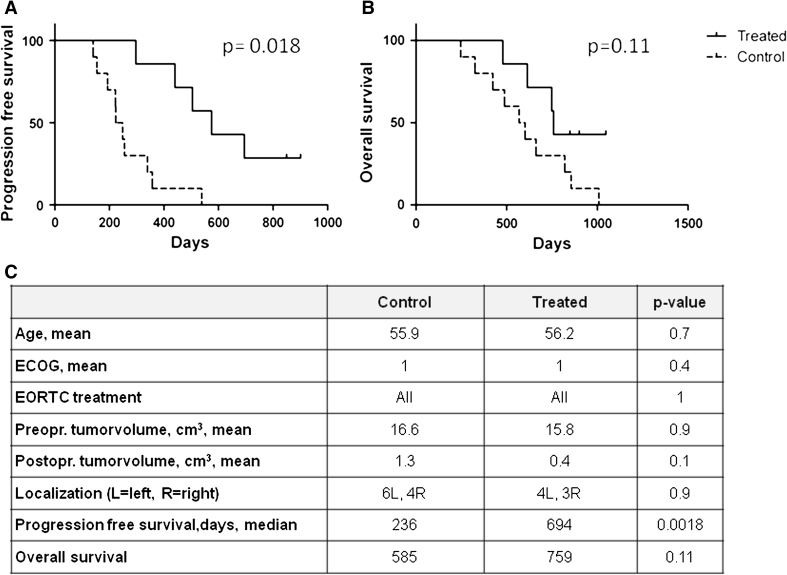
Survival of patient treated with DCs targeting GSCs compared to matched control patients treated with standard therapy. Comparison of the seven patients treated with DCs targeting GSCs compared to the ten controls matched by age, performance status, tumor volume, treatment modalities, and lack of corticosteroid treatment. **a** The vaccinated patients had a significantly longer progression-free survival (median of 694 days) compared to the matched controls (median 236 days; *p* = 0.0018, log-rank test). Two DC-treated patients had not developed recurrence (*short straight bars*). **b** The median overall survival was 759 days in the treated group compared to 585 days in the control group (*p* = 0.11, log-rank test). Three patients were still alive >1,000 days after surgery. **c** Descriptive data of the treated and control groups. Only PFS was significantly different between the two groups

Compared to the historical-matched controls, the groups were not significantly different in terms of important prognostic criteria. There was a trend toward larger tumor volumes in the controls and longer OS in the treated group (*p* = 0.1). The vaccinated patients had significantly longer PFS (median 694 days vs. 236 days, *p* = 0.0018, log-rank test, Fig. 3). Five of the treated patients developed tumor recurrence (at 10, 15, 17, 22, and 29 months, respectively). All patients in the matched control group experienced progression. Seven of these ten recurrences occurred earlier than the first recurrence in the vaccine group.

Six of the patients in the matched control group died earlier in the disease course than did the first patient in the treated group. The median OS from surgery was 759 days in the study group compared to 585 days in the matched control group (*p* = 0.11, log-rank test). Five of the seven patients were alive after 2 years. Three of the seven patients were alive after >1,000 days.

## Discussion

The present study describes the feasibility, safety, and potential efficacy of an active immunotherapy targeting GSCs and is, to our knowledge, the first report of a therapy targeting a characterized population of CSCs in any solid tumor [3, 4, 7, 9, 11, 27, 34].

Stem cells may be enriched by several techniques, but the sphere-forming assay has been shown to allow propagation of stem-like cells from a variety of organs, tumors, and species [7]. We previously compared somatic neural stem cells derived from different parts of the adult human central nervous system and GSC [11, 28] and found a correlation between the grade of malignancy and sphere-forming ability [11]. Consistent with the data presented here (Suppl. Fig. 3), sphere-forming ability is observed to be a stronger negative prognostic indicator than other well-established factors such as patient age, performance status, and expression of Ki67 or CD133 [35, 36]. Because tumorsphere formation was necessary for inclusion in the present protocol, patients were actually included based on a negative prognostic indicator.

The use of tumorsphere cultivation for the enrichment of GSCs allows for the proliferation of such cells while maintaining their tumorigenic phenotype [27, 32]. Unlike CSCs from hematopoietic malignancies, no definite surface markers exist for the isolation of GSCs. Not even CD133, which is the most studied marker, is specific for GSC [7, 12, 27, 33, 37].

Glioblastomas are highly heterogeneous both within tumors and between individual patients [38]. The use of autologous GSC antigens may stimulate immunity against antigens unique to the patient. The use of an individualized therapeutic approach may be very important when targeting GSC, as tumors may be derived from a range of different progenitor cells [6, 39]. One limitation to our approach is the loss of potentially important antigens from the cell populations that were unable to proliferate under sphere-forming conditions. Although autologous CSC generation is technically possible, it is expensive and cumbersome.

In patients with glioblastoma, the immune response—and thus the potential effect of a vaccine—can be impeded by several factors, including the use of corticosteroids, chemotherapy, and the presence of residual tumor. For that reason, patients on corticosteroids were excluded from the present study. Due to the immunosuppressive effect of cancer cells, it has recently been recommended that therapeutic vaccines only be tested in settings with a low burden of disease [40]. Our data are consistent with previous experimental investigations documenting that the immunosuppressive effects of GSCs can be overcome and that GSCs can be recognized and killed by CD8^+^ cytolytic T cells, and NK cells in murine tumor models [22].

Lymphopenia has been suggested to benefit treatment response in melanoma patients [41]. Similarly, in a cancer vaccine trial in patients with advanced stage melanoma, we found that a telomerase-derived peptide vaccination in combination with a temozolomide maintenance regimen was feasible and yielded a higher frequency of immune response [42]. Note that, standard radio-chemotherapy not only allows the induction of a tumor-specific immune response [42, 43], but it also may work synergistically by facilitating antitumor immunity. The lymphopenia induced by temozolomide may induce homeostatic cascades allowing thymic-independent antigen-driven T cell proliferation through a reduced activation threshold and T cell differentiation directly into effector T cells capable of rapid and intense response to antigens [44]. The lymphocyte counts of the patients in the current study remained very low throughout the vaccination period (Suppl. Fig. 5), and this may have resulted in less-than-optimal immune responses. Although the median survival benefit achieved by temozolomide is moderate [1], we found no convincing arguments for excluding the chemotherapy from the protocol in this early phase study. In a future study, however, we may consider modification of the standard temozolomide regimen based on lymphocyte levels.

The use of dendritic cells loaded with mRNA has several advantages over other DC-based approaches. In contrast to tumor protein, mRNA can be amplified in vitro. Amplification allows for a relatively small cell source to be used for the production of a large number of antigen-loaded DCs [29]. In addition, the use of short-lived RNA constructs is safer than DNA, which may integrate into the DCs genome and introduce oncogenic activity into cells returned to the patient. Finally, previous research has shown that RNA outperforms DNA in DCs T-cell inductions and that loading with tumor RNA is superior to loading with lysate or fusion of tumor cells with DCs [45].

We did not observe any significant treatment-related adverse effects among our study patients. The adverse events reported were within the normal range of what would be expected from standard therapy. DCs transduced with antigens from CSCs might elicit immune responses against normal stem cells; therefore, to monitor for possible cross-reaction against neural stem cells, we performed regular ophthalmologic exams to identify the development of inflammatory reactions against such cells in the eye [46]. The induction of an immune response against stem-like cells could also result in an autoimmune cross-reaction against other populations of somatic stem cells. To investigate this possibility, we monitored the levels of hematopoietic stem cell-derived lineages, as well as symptoms from organs highly dependent on stem cells for cellular turnover, such as skin and the gastrointestinal tract. We found no evidence of cross-reactions to other populations of somatic stem cells.

A primary limitation associated with the present approach is the scarce amount of GSC material available for immunological monitoring of T cell responses following vaccination. The need to obtain sufficient amounts of mRNA for vaccine production was prioritized throughout the study, but sufficient material for immune monitoring was available in only four of the seven study patients. To compensate for this, hTERT and survivin antigens were added to the vaccine because pools of long overlapping peptides were available for us to perform in vitro testing of T cell responses toward these targets. This makes the interpretation of our clinical data more complicated because tumor growth may have been influenced both by T cells directed against antigens expressed in the GSCs and by T cells specific for hTERT and survivin. On the other hand, a vaccine composed of a combination of “universal” or general cancer antigens and patient-specific antigens may well be the best recipe for a clinically efficacious vaccine in the future. In the present study, we were able to detect T cell responses against both the patient’s own GSC-lysate as well as the two defined antigens.

To our knowledge, we present the first patients treated with immune therapy targeting autologous GSC antigens. A recent study reported using DCs loaded with a combination of six antigens, and three have been reported enriched in the GSC population [47, 48]. That report does not report on any adverse events but suggests a possible effect on survival comparable to the data presented here. Evaluating tumor response in a low-powered study has limitations. Although the control patients are closely matched to the treated patients, the use of historical controls makes it difficult to ensure that all variables that could affect outcomes are equally distributed. Changes in tumor volume could be due to the late effects of standard therapy and pseudo-progression. In addition, the usefulness of tumor volume measurement might not be relevant when targeting the CSC–progenitor cell population, as this may not reduce tumor bulk but instead might eliminate further tumorigenic potential [3]. There was a nonsignificant difference in post-operative tumor volume between the treated group and the control patients, which could indicate a possible benefit for the treated group. However, the effects presented here on PFS and tumor volume reduction after the induction of an immune response are consistent with a therapeutic effect. The present results will, however, allow for a randomized phase II study to take place.

In conclusion, we were able to induce a GSC-specific immune response without eliciting serious adverse reactions. Our results support the CSC hypothesis and indicate that targeting the CSC population may be therapeutically rewarding. The use of sphere-forming capability for the propagation and enrichment of CSCs is well-established in the glioblastoma. The technology for enriching such cells is transferable to a variety of tumors; therefore, the immunotherapy protocol presented here may be used as a model for targeting CSCs in other solid tumors.

## Electronic supplementary material

Below is the link to the electronic supplementary material.

Supplementary material 1 (PDF 378 kb)
