# Supplementary material for: Therapeutic vaccination against autologous cancer stem cells with mRNA-transfected dendritic cells in patients with glioblastoma
Source: Cancer Immunol Immunother. 2013 Jul 2;62(9):1499–509. doi: 10.1007/s00262-013-1453-3 (PMC3755221; doi:10.1007/s00262-013-1453-3)
Supplement: Supplementary file 1 — Supplementary material 1 (PDF 378 kb) [file 262_2013_1453_MOESM1_ESM.pdf]

Supplementary information

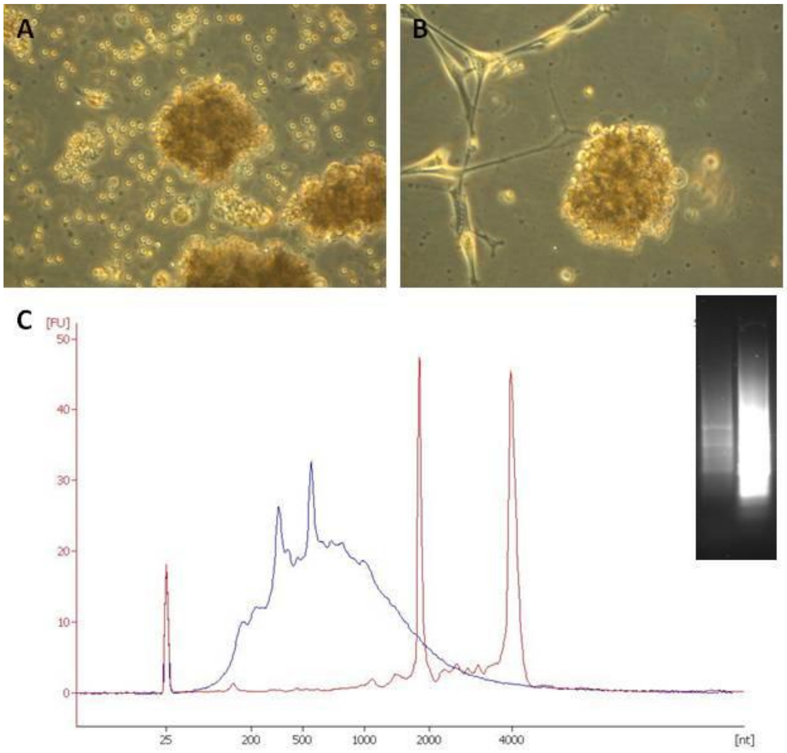

Suppl. Fig. 1: Tumorspheres and RNA quality

Cancer stem cells were cultivated under serum-free conditions producing primary (A), secondary, and tertiary (B) tumorspheres. Microelectrophoresis curves from total RNA (red) and amplified mRNA (blue) (C). Agarose gel electrophoresis under denaturing conditions display a smear present in total RNA (left) and amplified mRNA (right) (insert in C).

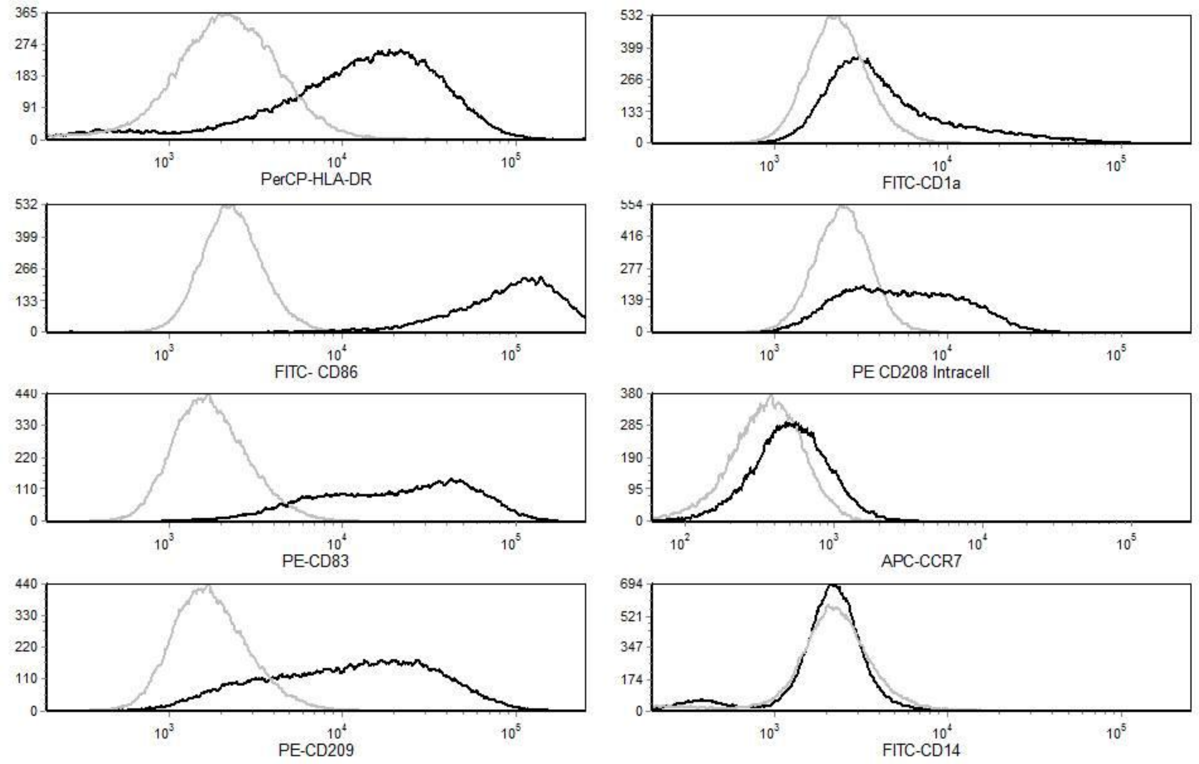

Suppl. Fig. 2: Flow cytometric analysis of *ex vivo* generated monocyte-derived dendritic cells in one representative patient

Specific antibody, dark curve. Isotype control, grey curve. Cells express high levels of HLA-DR in combination with the co-stimulatory CD86-molecule. Cells display a typical DC phenotype CD83+, CD208+, CD1a+, and CD209+. They contained both CD83- CD14- and CD83+ CD14- populations, where the CD83+ positive population was CD86+ and the CD83- were CD86dim. Some cells express the chemokine receptor CCR7+ present in a subset of mature DCs. Cells are monocyte marker CD14-.

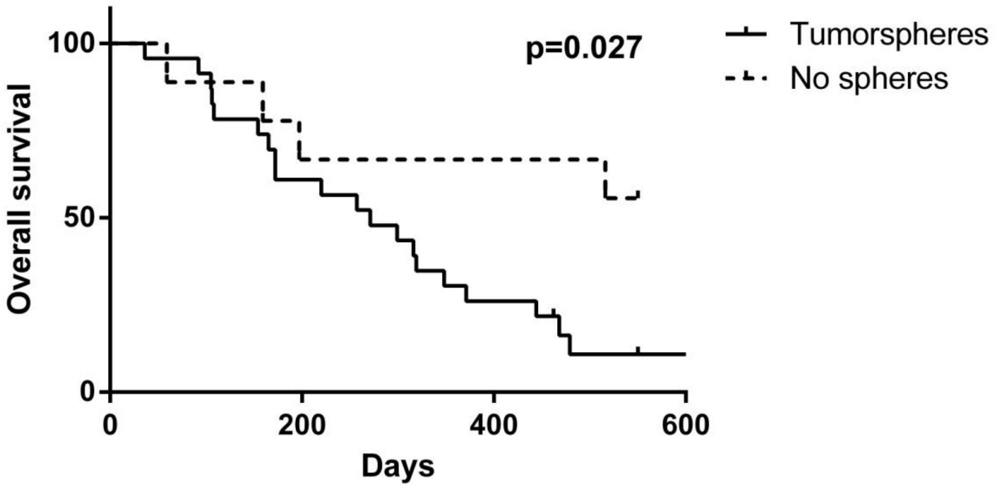

Suppl. Fig. 3: Overall survival according to biopsies ability to be propagated as tumorspheres

In a preclinical evaluation of a Good Manufacturing Procedure (GMP) transferable protocol biopsies from 32 glioblastoma patients were grown under serum-free, growth factor enriched conditions. In the 23 cultures able to produce tertiary tumorspheres during eight weeks, the median survival was 271 days, compared to a median not reached for those patients where cultures produced no tumorspheres (p=0.027, Log-rank test). For individual patient data please see Suppl. Table 2.

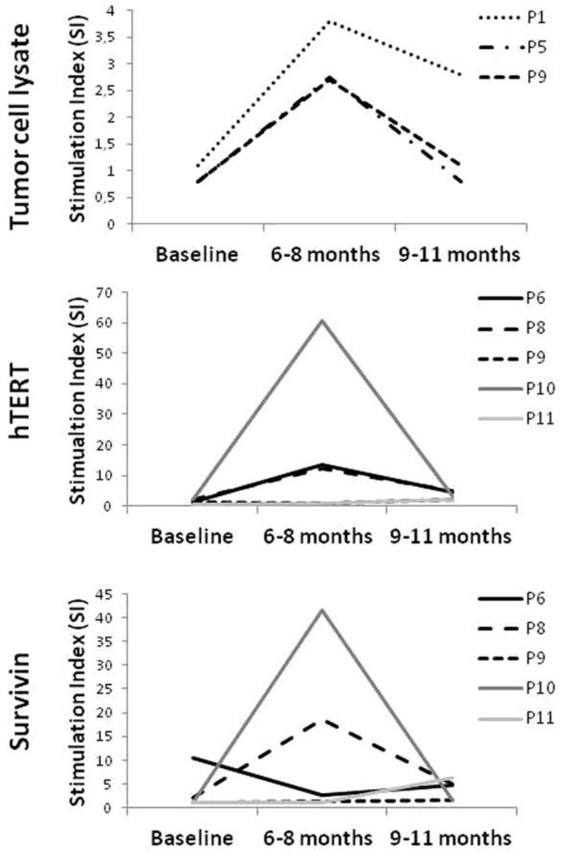

Suppl. Fig. 4: Immune response evaluated by lymphocyte proliferation upon stimulation by tumorsphere lysate (top) or a mixture of peptides from hTERT (middle) or surviving (bottom)

Peripheral blood lymphocytes were harvested before surgery, during vaccination (6-8 months) and at the end of vaccination period (9-11 months). All patients developed a significant T-lymphocytes proliferation response induced by tumorsphere lysate (TSL), hTERT or survivin peptide *in vitro*. In patient #6, #8, #10 and #11 there were not enough tumorsphere cellular material to allow for testing of induced T-lymphocyte proliferation. hTERT- and survivin-mRNA transfected DCs was added to the treatment from patient #6. Please refer to Table 2 for numeric values.

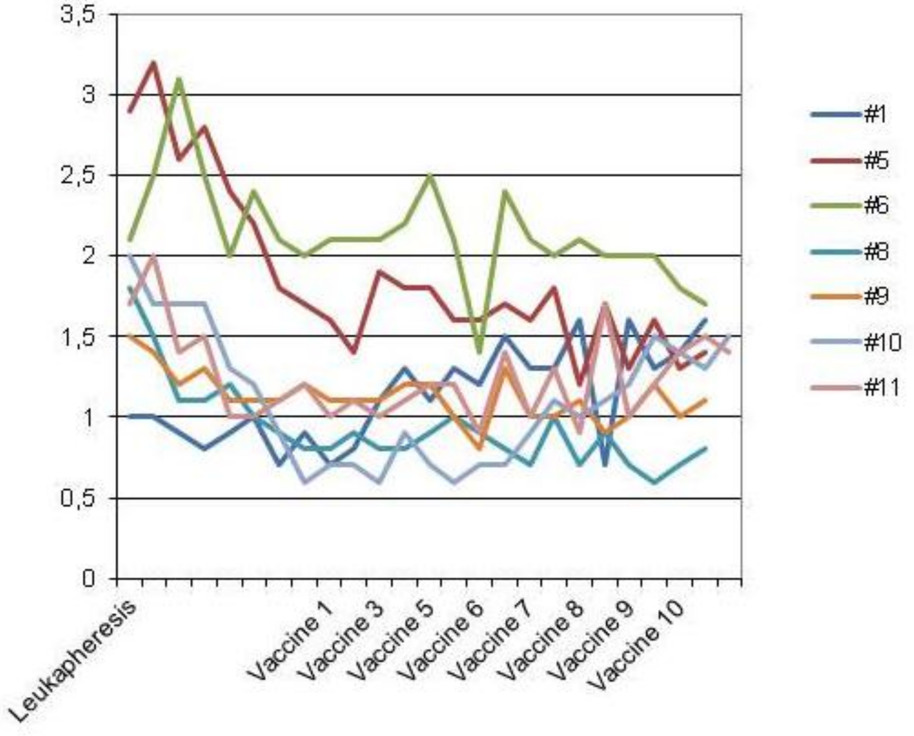

Suppl. Fig. 5: Lymphocyte levels in vaccinated patients

In the seven presented patients lymphocyte levels were below 1.1 x 10^9 cells/ml, considered lymphopenic, at 29 of 70 time points of the ten first vaccinations.

#	Age, sex	RPA	ECOG	Preop Tumor Vol	Postop Tumor Vol	PFS days	OS days
1	49. M	3	0	6.2	0	223	662
2	56. F	4	0	26.8	0	140	820
3	56. M	4	1	26.0	0	154	1009
4	54. M	4	1	10.7	0	248	569
5	68. F	4	1	0.9	0.3	222	424
6	49. F	3	1	18.8	1.6	255	325
7	63. F	4	1	5.3	1.7	193	246
8	48. M	3	1	19.9	1.8	357	488
9	60. F	4	1	38.5	3.0	339	980
10	59. M	4	1	12.6	4.7	538	601

Suppl. Table 1: Historical control patients characteristics

Abbreviations: F; female. M; male. RPA; Recursive partitioning analysis group for prognostic differentiation according to the Radiation Therapy Oncology Group (RTOG) (31). NA; not applicable.

Pat #	Age	ECOG	# surgery	Resection grade	Survival	Diagnosis
**Sphere forming**						
1	60	2	1	ST	299	GBM. primary
2	51	2	1	ST	316	GBM. primary
3	65	1	1	ST	659	GBM w/ Oligo component
4	77	3	1	ST	154	GBM. primary
5	71	2	1	ST	271	GBM. primary
6	73	2	1	ST	92	GBM. primary
9	82	2	1	ST	108	GBM. primary
10	60	3	2	ST	106	GBM. secondary
12	59	1	1	ST	528	GBM. primary
15	48	3	1	ST	444	GBM. primary
16	48	4	1	ST	319	GBM. primary
17	76	3	1	ST	172	GBM. primary
18	84	0	1	T	257	GBM. primary
19	61	1	1	T	468	GBM. primary
21	45	0	2	T	462	GBM. secondary
24	69	3	1	ST	220	GBM. primary
25	65	3	1	ST	36	GBM. primary
26	63	1	1	T	479	GBM. primary
27	62	1	1	T	371	GBM. primary
28	62	1	1	ST	105	GBM. primary
29	60	2	1	ST	165	GBM. primary
30	56	1	1	ST	172	GBM. primary
32	81	1	2	ST	348	GBM. primary
**Non-sphere forming**						
7	68	3	1	ST	159	GBM. primary
8	67	3	1	ST	NA	GBM. primary
11	59	1	2	T	NA	GBM. primary
13	62	2	1	ST	NA	GBM. primary
14	62	2	1	T	516	GBM. primary
20	57	2	1	ST	197	GBM. primary
22	27	2	2	ST	NA	GBM. secondary
23	63	1	1	ST	NA	GBM. primary
31	80	2	1	ST	59	GBM. primary

Suppl. Table 2: Clinical data on 34 consecutive patients with glioblastoma where biopsies tested for sphere forming ability
